# Pharmacokinetics of chloroquine and primaquine in healthy volunteers

**DOI:** 10.1186/s12936-021-04035-z

**Published:** 2022-01-08

**Authors:** André Daher, Douglas Pereira Pinto, Laís Bastos da Fonseca, Heliana Martins Pereira, Diego Medeiros Dias da Silva, Letícia de Sá Fernandes Vallim da Silva, Alessandra Lanzillotta Esteves, Juliana J. Soares Medeiros, Jorge Souza Mendonça

**Affiliations:** 1 Vice-Presidency of Research and Biological Collections, Oswaldo Cruz Foundation (FIOCRUZ), Avenida Brasil, 4036, Manguinhos, Rio de Janeiro, Brazil; 2 Laboratory of Pharmacokinetics (SEFAR), Oswaldo Cruz Foundation (FIOCRUZ), Rio de Janeiro, Brazil; 3 Institute of Drug Technology (Farmanguinhos), Oswaldo Cruz Foundation (FIOCRUZ), Rio de Janeiro, Brazil

**Keywords:** Malaria, Vivax, *Plasmodium vivax*, Chloroquine, Primaquine, Pharmacokinetics

## Abstract

**Background:**

Vivax malaria is a neglected disease. There is an irrefutable need for better treatments with higher acceptability and efficacy. The treatment efficacy is influenced by many factors, including bioavailability. Hence, a straightforward strategy to improve vivax malaria treatment efficacy is the deployment of good quality formulations of primaquine and chloroquine. As these treatments were developed more than 70 years ago, many of the available data on blood levels of both drugs are based on obsolete analytical methodologies or pharmaceutical formulations, which are not available anymore. Herein, the results of three bioequivalence studies are presented, providing individual pharmacokinetic data on chloroquine and primaquine of more than a hundred healthy volunteers and using up-to-date analytical methods.

**Methods:**

Three trials were designed as a single centre, randomized, single dose, open label, fasting, crossover bioequivalence studies comparing a new coated chloroquine tablet to the uncoated tablet, and 5 and 15 mg primaquine formulations to either an international reference product or the currently distributed tablets. Plasma concentrations of chloroquine and primaquine were measured using a validated HPLC–MS/MS method in accordance with current international regulatory requirements for bio-analytical methods.

**Results:**

In total, a hundred eleven healthy volunteers of both genders were included in the three studies (n = 32; 30 and 56 respectively). No serious adverse events occurred. Drugs levels were measured in 5,520 blood samples. The estimated ratio of the geometric means of Cmax, AUC0-t and AUC0-inf of test and reference drugs and their 90% CI for chloroquine 150 mg, primaquine 15 mg and primaquine 5 mg were: 95.33% (89.18; 101.90), 86. 85% (82.61; 91.31), and 84.45% (76.95; 92.67); 93.28% (81.76; 106.41), 94.52% (86.13; 103.73) and 93.93% (85.83; 102.79); 97.44% (90.60; 104.78), 93.70% (87.04; 100.87) and 91.36% (85.27; 97.89), respectively. As Cmax and AUC0-t 90% CI were within the acceptance interval of 80–125% in all cases, the formulations tested were bioequivalent.

**Conclusions:**

In conclusion, the three studies provided detailed chloroquine and primaquine pharmacokinetic data in accordance with current regulatory standards. Together with other open data initiatives, this individual data may increase the accuracy of pharmacokinetic models guiding best dose, new combinations, regimens and formulations to optimize the current chloroquine and primaquine treatments for vivax malaria. The data presented here may support the deployment of high-quality drugs and evidence-based public health policies.

**Supplementary Information:**

The online version contains supplementary material available at 10.1186/s12936-021-04035-z.

## Background

Vivax malaria is not listed as one of the seventeen Neglected Tropical Diseases by the World Health Organization (WHO) [1] However, there has been recent international agreement that the importance of vivax malaria has been neglected [2–5] and there is an irrefutable need for better treatments [6] with higher acceptability, efficacy and a better safety profile. Whilst the screening and design of new molecules is important, incremental innovations may also have a place. Indeed, the Medicines for Malaria Venture (MMV) Target Product Profile (TPP) starts with optimizing current treatments [7], which have not changed since the 50’s [4]. In most of the world, the first-line treatment recommendation for vivax malaria is chloroquine and primaquine.

Chloroquine (Resochin) was discovered in 1934 by Germans researchers, however it was named chloroquine in the USA during WWII [8]. Chloroquine has a long record of human use. It has been one of the most successful drugs deployed against malaria and it is still the main blood schizonticidal drug to treat malaria vivax in most parts of the world, including Brazil. By the 1980s, hundreds of millions of chloroquine malaria treatments were used worldwide annually [9], > 190 tones/year in Africa alone. In 1985, the global production of chloroquine reached 1300 tones/year [10]. The optimal dose should be given to ensure the best efficacy of this drug and chloroquine is commonly under-dosed in the treatment of vivax malaria, especially in children younger than 5 years [11]. The selection of the best dose in children, as well as the decision on new combinations, regimens, formulations, and models to optimize the current treatments should be based on drug exposure.

Primaquine, an 8-aminoquinoline, was first made available in the 1950s [12] to soldiers returning from the Korean war [13]. Primaquine affects mainly the hepatic forms of *Plasmodium vivax* that cause relapses, but it is also active against blood stages of the parasite [14]. Relapses are a major contribution to the global *P. vivax* burden and a central target to improve the efficacy of the radical treatment for vivax malaria. Primaquine efficacy depends on the immune status of the patients, CYP2D6 activity, body mass index, partner drug, drug-drug interaction, differences in the biology of the parasite, hypnozoite load, but also on adherence and bioavailability [15]. Impaired bioavailability may be due to poor quality medicines and this is a neglected public health problem [16] that is relevant for all anti-malarials. As these drugs were developed more than 70 years ago, most of the available data on blood levels are based on obsolete analytical methodologies and pharmaceutical formulations which are no longer readily available.

Herein the results of three bioequivalence studies are presented providing individual pharmacokinetic data (PK) on chloroquine and primaquine of more than a hundred health volunteers, using up-to-date analytical methods. These randomized cross over studies explored the pharmacokinetic profile of: (1) a newly produced coated chloroquine tablet compared to the uncoated tablet previously distributed by the Brazilian National Health System (SUS) and (2) primaquine 5 mg and 15 mg tablets compared to either the currently distributed tablet or the international reference product. The studies were conducted to comply with national regulatory requirements in Brazil informing drug exposure levels in healthy volunteers. The results provide reliable individual data which may be useful for pharmacokinetic modelling of both drugs and may contribute to improvements of vivax malaria treatments.

## Methods

### Studies designs, populations and procedures

Three bioequivalence studies were conducted at Hospital Itatiba, Itatiba, São Paulo, which is accredited by the Brazilian Regulatory Agency (ANVISA). The first study (study1_Cq) compared a new coated chloroquine tablet to the uncoated tablet that was previously distributed by the Brazilian National Health System. The second study (study2_Pq5&15 mg) compared one tablet of primaquine 5 mg or 15 mg to an international reference primaquine 15 mg tablet. The third study (study3_Pq5mg) compared three tablets of primaquine 5 mg to the primaquine 15 mg tablet currently distributed.

Two trials (study1_Cq and study3_Pq5mg) were designed as a single centre, randomized, single dose, open label, fasting, two-phase, two-sequences, crossover bioequivalence study. The second study (study2_Pq5&15 mg) was designed as a single centre, randomized, single dose, open label, fasting, three-phases, six-sequences, crossover bioequivalence study.

All trials had the same inclusion and exclusion criteria for subjects. Eligible healthy volunteers willing to sign the informed consent were enrolled in the study if they met the following inclusion criteria: age between 18 and 50 years, Body-Mass-Index (BMI) between 18.5 and 29.9 kg/m^2^, and normal results at the physical examination and clinical laboratory tests (haematology, biochemistry, urinalysis, HIV and Hepatitis B and C; parasitological faeces test, a 12-lead electrocardiogram (ECG)) and a negative β-HCG for the female subjects before each administration of the study drugs. The exclusion criteria were: known hypersensitivity to chloroquine or primaquine, use of any concomitant medicines, health problems, alcohol or drug abuse. In the first and third trials (study1_Cq and study3_Pq5mg), included subjects were randomly allocated to one of the two study treatment sequences (RT or TR). In the second trial (study2_Pq5&15 mg) included participants were randomly allocated to one of the six study treatment sequences (RT2T1, T1RT2, T2T1R, T1T2R, T2RT1 or RT1T2). The test formulation as well as the reference formulation were administered under fasten conditions. Blood samples (7.5 ml each) were drawn into EDTA K3 tubes and stored at − 70 °C (± 10 °C) for pharmacokinetics evaluation. Adverse events were assessed during hospitalization, at 10, and at 30 days after the administration of the drugs.

For the first study (study1_Cq), blood sampling schedule was 0 (pre-dose 30 min before dosing), 00 h 30 min, 01 h 00 min, 01 h 30 min, 02 h 00 min, 02 h 30 min, 03 h 00 min, 03 h 30 min, 04 h 00 min, 04 h 30 min, 05 h 00 min, 05 h 30 min, 06 h00 min, 06 h 30 min, 07 h 00 min, 07 h 30 min, 08 h 00 min, 09 h 00 min, 11 h 00 min, 24 h00 min, 36 h min, 48 h 00 min, 72 h 00 min (truncated) after oral administration of each treatment. The wash out period was 21 days. This study design considered that the chloroquine half-life is longer than 72 h and used the truncated 72 h sampling schedule [17, 18].

The second and third (study2_Pq5&15 mg and study3_Pq5mg) primaquine studies blood sampling was conducted as follows: 0 (pre-dose 30 min before dosing), 00 h 15 min, 00 h 30 min, 00 h 45 min, 01 h 00 min, 01 h 15 min, 01 h 30 min, 01 h 45 min, 02 h 00 min, 02 h 20 min, 02 h 40 min, 03 h 00 min, 03 h 20 min, 03 h 40 min, 04 h 00 min, 04 h 30 min, 05 h 00 min, 06 h 00 min, 08 h 00 min, 10 h 00 min, 12 h 00 min, 24 h 00 min. The wash out period was 14 days.

Concentrations of both drugs were measured after liquid–liquid extraction using the Methyl tert-butyl ether (MTBE) and analysis by HPLC–MS/MS. The chloroquine and primaquine concentration range established for the method was 0.25 to 100.00 and 0.50 to 200.00 ng/mL, respectively. Both methods are validated in accordance with Brazilian [19] and international regulatory requirements for bio-analytical methods [20]. The pharmacokinetics assays were conducted at the Laboratory of Pharmacokinetics (SEFAR)/Oswaldo Cruz Foundation, which is accredited by ANVISA.

Using a non-compartmental model, the PK parameters estimated were maximum concentration (Cmax), Area Under de Curve (AUC) 0-t (until 24/72 h), AUC0-inf, time to maximum concentration (Tmax), half-life (T1/2) and constant of elimination (Kel). The 90% confidence interval (CI) for each parameter was calculated, as the variance analysis and the power of the test using the software SAS® 9.4, SAS Institute Inc. considering the 5% significance level and power of 80% at the Schuirmann test. Sample size was calculated based on literature and previous studies [21] using PROC POWER (SAS® 9.4) and allowing for 20% loss to follow-up. The main endpoints were the 80 –125% interval for Cmax and ASC0-t (log transformed data), as required by regulatory agencies. This approach comprises the calculation of a 90% CI for the ratio of the averages (population geometric means) of the PK measures for the Test and Reference [17, 18].

### Ethics statement

The three clinical study protocols and informed consents were reviewed and approved by the Ethics Committee at the University of Jaguariuna (CAAE 67860317.3.0000.5409, CAAE 33704720.5.0000.5599, CAAE 09981119.5.0000.5599). This committee is accredited by the Brazilian National Council on Ethics in Research (CONEP)/Ministry of Health. The clinical study was conducted in accordance with Good Clinical Practice [22, 23]. Written informed consent was obtained for every subject prior to enrolment. All subjects were informed about the nature of the trial and possible risks bound to it, and that they were free to withdraw their consent and discontinue participation at any time. The study investigators and staff observed the confidentiality of the records. The trials were registered at ANVISA in the National Registry for Equivalence and Bioequivalence Studies (SINEB) and at the Brazilian Clinical Trials Registry (REBEC RBR-6mjqrf, RBR-27qyp2, RBR-105nyj86).

## Results

### Coated chloroquine vs. reference chloroquine (study1_Cq)

Thirty-two healthy volunteers (16 males and 16 females) were included in the first study (study1_Cq). One volunteer withdrawn the informed consent and was not included in the final analyses. No serious adverse events occurred during the trial. Twenty seven adverse events were recorded. One AE (mild headache) was possibly related to the study drugs.

**Fig. 1:**
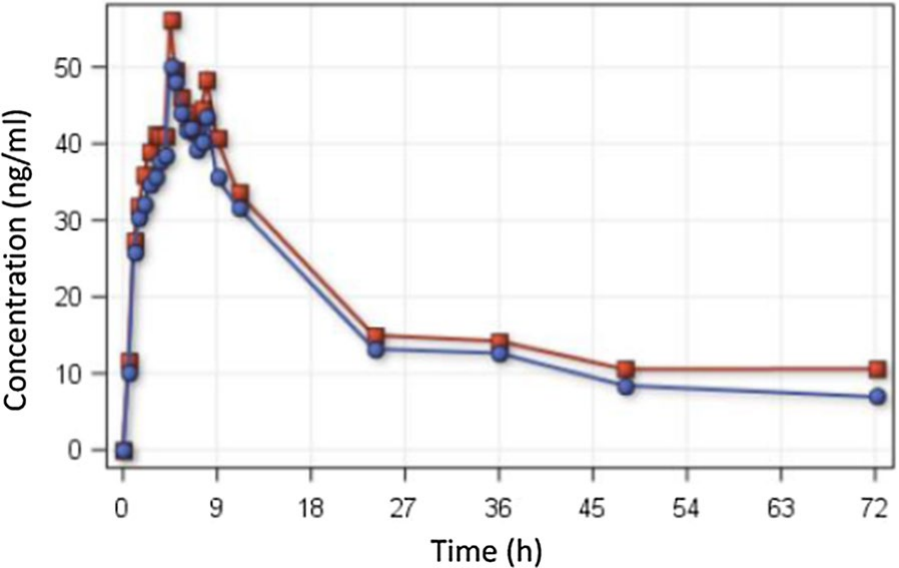
Chloroquine pharmacokinetic curve. Curves of mean concentration (ng/ml) versus time (hours). Blue: coated chloroquine 150 mg, red: reference chloroquine 150 mg

CQ levels were measured in 1421 samples using a validated HPLC–MS/MS method. The estimated ratio of the geometric means of C_max_, AUC_0-t_ and AUC_0-inf_ of the test and of the reference drugs and their 90% CI were 95.33% (89.18; 101.90); 86. 85% (82.61; 91.31); and 84.45% (76.95; 92.67), respectively. The curves of mean concentration (ng/ml) versus time (hours) are presented in Fig. 1.

The individual concentrations of all plasma samples for each volunteer are available (n = 1421) at the data sharing platforms WorldWide Antimalarial Resistance Network (WWARN) and ARCA Dados/ Fiocruz (https://doi.org/10.35078/F8RSR9) where the final report including analytical methodology validation and detailed statistical analysis (in Portuguese) are available. Detailed pharmacokinetics parameters are presented in the Additional file 1: Table S1.

The estimated ratio of the geometric means of C_max_, AUC_0-t_ of the test formulation and of the reference drug and their 90% CI were within the acceptance interval of 80–125%. Thus, the chloroquine formulations compared were bioequivalent.

### Primaquine results

#### Primaquine 15 mg and primaquine 5 mg vs. reference primaquine 15 mg (study2_Pq5&15 mg)

In the study2_Pq5&15 mg, thirty health volunteers (15 males and 15 females) were included. Two volunteers withdrawn the informed consent and were excluded of the final analysis.

Primaquine levels were measured in 1848 samples from 28 participants. The estimated ratio of the geometric means of C_max_, AUC_0-t_ and AUC_0-inf_ of primaquine 15 mg test and reference drug (15 mg) and their 90% CI were 93.28% (81.76; 106.41); 94.52% (86.13; 103.73) and 93.93% (85.83; 102.79), respectively.

**Fig. 2:**
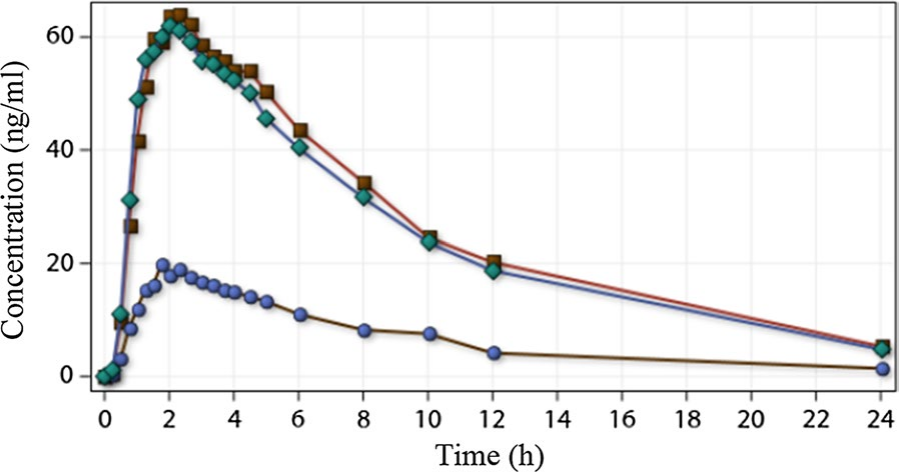
Primaquine 15 mg and 5 mg pharmacokinetic curve. Curves of mean concentration (ng/ml) versus time (hours). Blue: primaquine 5 mg, green: primaquine 55 mg, red: reference primaquine 15 mg

As dose and pharmacokinetics parameters linearity was expected, the concentration of each sample point of the 5 mg arm were multiplied by three before the statistical analysis. The estimated ratio of the geometric means of C_max_, AUC_0-t_ and AUC_0-inf_ of primaquine 5 mg test and reference drug (15 mg) and their 90% CI were 93.07% (81.61; 106.15); 74.14% (67.57; 81.35) and 74.24% (67.86; 81.23), respectively. The individual concentrations of plasma samples (n = 1848) are available at the data sharing platforms WWARN and ARCA Dados/Fiocruz. Both 5 mg and 15 mg curves of mean concentration (ng/ml) versus time (hours) are presented in Fig. 2. Further pharmacokinetics parameters are presented in the Additional file 2: Table S2.

The estimated ratio of the geometric means of C_max_, AUC_0-t_ of primaquine 15 mg test formulation and the reference formulation and their 90% CI were within the regulatory approval interval of 80–125%. Hence the 15 mg formulations were bioequivalent. Conversely, the primaquine 5 mg test formulation did not meet the acceptance criteria and it was deemed not bioequivalent. Therefore, a third study was conducted to compare one tablet of primaquine 15 mg formulation with 3 tablets of the new primaquine 5 mg.

#### Primaquine 5 mg (03 tablets) vs. reference primaquine 15 mg (study3_Pq5mg)

Fifty six healthy volunteers (28 males and 28 females) were included in this study (study3_Pq5mg). Two volunteers withdrawn their informed consent, one volunteer tested positive for drug abuse and another tested positive for SARS-CoV-2. The final analyses were conducted with 52 participants. No serious adverse events occurred during the trial. Twenty two adverse events were documented. Eleven AEs (mild headache and laboratory abnormal results) were either possibly or probably related to the study drug.

**Fig. 3:**
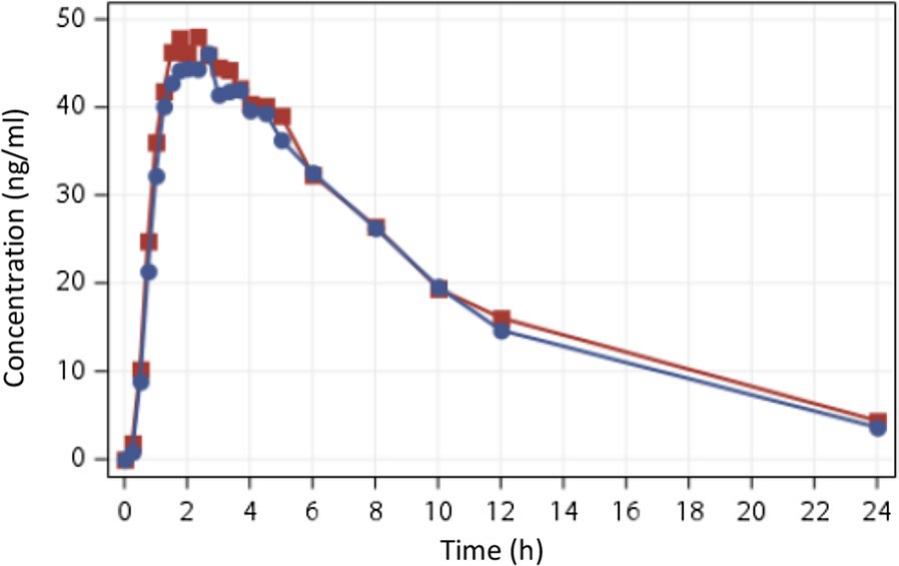
Primaquine 5 mg, 3 tablets pharmacokinetic curve. Curves of mean concentration (ng/ml) versus time (hours). Blue: primaquine 5 mg (03 tablets), red: reference primaquine 15 mg

2245 samples were analysed to measure primaquine levels, each. The estimated ratio of the geometric means of C_max_, AUC_0-t_ and AUC_0-inf_ of three tablets of primaquine 5 mg test formulation and reference primaquine (15 mg) and their 90% CI were 97.44% (90.60; 104.78); 93.70% (87.04; 100.87); and 91.36% (85.27; 97.89) respectively. These results comply with regulatory requirements, thus bioequivalence was established. The curves of mean concentration (ng/ml) versus time (hours) are presented in Fig. 3. Detailed pharmacokinetics parameters are presented in the Additional file 3: Table S3.

## Discussion

The vivax malaria treatment efficacy is influenced by previous exposure to the parasite (immune status), metabolic impairment (CYP2D6 activity), age (body mass index), partner drug used (that can be either artemisinin-based combination or chloroquine), but also by adherence and bioavailability. Poor quality medicines lead to unpredictable bioavailability and drug exposure. This is a neglected public health problem that is relevant for vivax malaria treatments. Hence a straightforward strategy to improve the efficacy of those treatments is the deployment of good quality formulations of primaquine and chloroquine with known bioavailability.

This study demonstrated that three new manufactured formulations in Brazil were bioequivalent to their comparators. However, the 5 mg formulation failed to show bioequivalence in the second study (study2_Pq5&15 mg). This may be due to the statistical correction of the plasma concentration of the primaquine 5 mg study arm, meaning that this result might have been a statistical artefact, including the effect of a high number of sample points with very low concentrations. This result may also challenge the primaquine dose/drug exposure linearity paradigm. As stringent regulatory agencies have well defined bioequivalent statistical analysis procedures [18], a new study was conducted using the same batch. The new study design comparing 3 tablets of primaquine 5 mg with one tablet of 15 mg was proven to be a valid approach and the bioequivalence was confirmed.

The relation of dose and drug exposure depends on the pharmaceutical formulation. As chloroquine and primaquine regulatory approval dates back to 70 year ago, most of pharmacokinetics parameters available in the literature are supported by pharmaceutical products manufactured with technologies no longer in use or by out-of-date analytical methods. The pharmacokinetics parameters using up-to-date analytical methods on the available formulations is still a knowledge gap, or at least undisclosed industrial information. Defining the proper exposure to chloroquine and primaquine have further struggles. These drugs are not included in the WHO reference drug list. As the WHO refrains from defining comparators that are valid worldwide, this study used the FDA listed reference product as a comparator, however it is neither produced nor widely used in the USA. The new coated chloroquine 150 mg and primaquine 15 mg formulations are now listed as reference drugs by the Brazilian national regulatory agency. The individual patient data of this manuscript was shared and may help future research and address these current knowledge gaps.

Poor adherence is also an important cause of effectiveness decay [24] that can be partly tackle by pharmaceutical technology. The new coated chloroquine formulation was developed to mask the bitter taste and improve acceptability and adherence of the malaria vivax treatment. However the paediatric population needs were not totally addressed, as the most children-friendly primaquine 5 mg formulation should be dispersible and mask the flavour. The lack of adherence and under dosing of this population might be the cause of high rates of relapse [11, 25] and must be addressed in future pharmaceuticals developments to avoid the endurance of reservoirs. The vivax malaria treatment still needs improvement, specially focusing in paediatric population.

The studies reported in this manuscript have some limitations. As these studies were conducted in healthy populations, this may reduce the validity of the results in paediatric and vivax malaria infected population, since the drug PK may be disrupted by the disease and by the patient BMI. Also, these studies evaluated a single dose administration and, drug exposure may be higher during the three days treatment of chloroquine, a long half-life drug. Pooled analysis of individual patient data from thriving clinical trial data repositories, as WWARN [26] and ARCA Dados/Fiocruz may help to define proper drug exposure in vivax infected patients and paediatric populations.

## Conclusion

These studies provided detailed chloroquine and primaquine PK data according to current regulatory standards. Together with other open data initiatives, this individual data may increase the accuracy of PK models guiding best dose, new combinations, regimens and formulations to optimize the current chloroquine and primaquine vivax malaria treatments. The data presented here may support the deployment of high quality drugs and evidence-based public health policies.

## Supplementary Information

**Additional file 1: Table S1.** Chloroquine 150 mg pharmacokinetics parameters (n = 31) *(study1_Cq)*.

**Additional file 2: Table S2.** Primaquine 05 and 15 mg pharmacokinetics parameters (n = 28) *(study2_Pq5&15 mg)*.

**Additional file 3: Table S3.** Primaquine 05 mg pharmacokinetics parameters (n = 52) *(study3_Pq5mg)*.

## Data Availability

Detailed pharmacokinetics parameters are available as supplementary materials. The drug concentrations at every collected point available is provided in the clinical trial data repositories WWARN and ARCA Dados/Fiocruz.

## Abbreviations

AE: Adverse events
ANVISA: Brazilian National Regulatory Agency
AUC: Area Under de Curve
CI: Confidence interval
Cmax: Maximum concentration
CNS: Brazilian National Health Council
CQ: Chloroquine
ICTRP: International Clinical Trials Registry Platform
HPLC–MS/MS: High performance liquid chromatography-tandem mass spectrometry
Kel: Constant of elimination
SD: Standard deviations
R: Reference drug
SEFAR: Equivalence and Pharmacokinetics laboratory
T: Test drug
T1/2: Half-life
Tmax: Time to maximum concentration
WWARN: WorldWide Antimalarial Resistance Network
