# Supplementary material for: Pharmacokinetics of chloroquine and primaquine in healthy volunteers
Source: Malar J. 2022 Jan 8;21:16. doi: 10.1186/s12936-021-04035-z (PMC8742557; doi:10.1186/s12936-021-04035-z)
Supplement: Supplementary file 1 — Additional file 1: Table S1. Chloroquine 150 mg pharmacokinetics parameters (n = 31) (study1_Cq). [file 12936_2021_4035_MOESM1_ESM.docx]

# **Additional Material 1.**

# **Table S1. Chloroquine 150 mg pharmacokinetics parameters (n=31) *(study1_Cq)***

|  | **AUC 0-t** | **AUC 0-inf** | **Cmax** |
| --- | --- | --- | --- |
| **Geometric mean Test** | 1,081.77 | 1,420,61 | 54,01 |
| **Geometric mean Reference** | 1,245.50 | 1,682,29 | 56,65 |
| **Ratio T/R (%)** | 86.85 | 84.45 | 95.33 |
| **CI 90%** | (82.61; 91.31) | (76.95; 92.67) | (89.18; 101.90) |
| **CV (%)** | 11.64 | 21.43 | 15.53 |
