# Supplementary material for: Pharmacokinetics of chloroquine and primaquine in healthy volunteers
Source: Malar J. 2022 Jan 8;21:16. doi: 10.1186/s12936-021-04035-z (PMC8742557; doi:10.1186/s12936-021-04035-z)
Supplement: Supplementary file 2 — Additional file 2: Table S2. Primaquine 05 and 15 mg pharmacokinetics parameters (n = 28) (study2_Pq5&15 mg). [file 12936_2021_4035_MOESM2_ESM.docx]

# **Additional Material 2.**

# **Table S2. Primaquine 05 and 15 mg pharmacokinetics parameters (n=28) *(study2_Pq5&15mg)***

|  | **AUC 0-t** | **AUC 0-inf** | **Cmax** |
| --- | --- | --- | --- |
| **Geometric mean primaquine 5mg (T1)** | 441.36 | 477.06 | 65.19 |
| **Geometric mean primaquine 15mg**  **(T2)** | 562.71 | 603.54 | 65.33 |
| **Geometric mean Reference** | 595.31 | 642.55 | 77.04 |
| **Ratio T1/R (%)** | 74.14 | 74.24 | 93.07 |
| **CI 90%** | (67.57; 81.35) | (67.86; 81.23) | (81.61; 106.15) |
| **Ratio T2/R (%)** | 94.52 | 93.93 | 93.28 |
| **CI 90%** | (86.13; 103.73) | (85.83; 102.79) | (81.76; 106.41) |
| **CV (%)** | 20.96 | 20.30 | 30.02 |
