# Supplementary material for: Pharmacokinetics of chloroquine and primaquine in healthy volunteers
Source: Malar J. 2022 Jan 8;21:16. doi: 10.1186/s12936-021-04035-z (PMC8742557; doi:10.1186/s12936-021-04035-z)
Supplement: Supplementary file 3 — Additional file 3: Table S3. Primaquine 05 mg pharmacokinetics parameters (n = 52) (study3_Pq5mg). [file 12936_2021_4035_MOESM3_ESM.docx]

# **Additional Material 3.**

# **Table S3. Primaquine 05 mg pharmacokinetics parameters (n=52) *(study3_Pq5mg)***

|  | **AUC 0-t** | **AUC 0-inf** | **Cmax** |
| --- | --- | --- | --- |
| **Geometric mean Test** | 385.13 | 435.10 | 49.48 |
| **Geometric mean Reference** | 411.02 | 476.24 | 50.79 |
| **Ratio T/R (%)** | 93.70 | 91.36 | 97.44 |
| **CI 90%** | (87.04; 100.87) | (85.27; 97.89) | (90.60; 104.78) |
| **CV (%)** | 22.70 | 20.99 | 22.37 |
